# Specificity of synapse formation in *Aplysia*: paracrine and autocrine signaling regulates bidirectional molecular interactions between sensory and non-target motor neurons

**DOI:** 10.1038/s41598-020-62099-4

**Published:** 2020-03-23

**Authors:** Anamaria Alexandrescu, Thomas James Carew

**Affiliations:** 1 Neuroscience Institute, New York University Langone Medical Center, New York, New York, 10016 USA; 2 Center for Neural Science, New York University, New York, New York, 10003 USA

**Keywords:** Neuroscience, Development of the nervous system, Learning and memory, Molecular neuroscience

## Abstract

The formation of appropriate neural connections during development is critical for the proper wiring and functioning of the brain. Although considerable research suggests that the specificity of synapse formation is supported by complex intercellular signaling between potential presynaptic and postsynaptic partners, the extracellular factors and the intracellular signal transduction pathways engaged in this process remain largely unknown. Using the sensory-motor neural circuit that contributes to learning in defensive withdrawal reflexes in *Aplysia californica*, we investigated the molecular processes governing the interactions between sensory neurons and both target and non-target motor neurons during synapse formation in culture. We found that evolutionarily-conserved intercellular and intracellular signaling mechanisms critical for learning-related plasticity are also engaged during synaptogenesis in this *in vitro* model system. Our results reveal a surprising bidirectional regulation of molecular signaling between sensory neurons and non-target motor neurons. This regulation is mediated by signaling via both paracrine and autocrine diffusible factors that induce differential effects on transcription and on protein expression/activation in sensory neurons and in target and non-target motor neurons. Collectively, our data reveal novel molecular mechanisms that could underlie the repression of inappropriate synapse formation, and suggest mechanistic similarities between developmental and learning-related plasticity.

## Introduction

The formation of specific neural connections is critical for the proper development and function of the nervous system. Specificity of synapse formation is supported by complex bidirectional intercellular and intracellular signaling between and within potential pre- and postsynaptic partners^1^. The molecules mediating these intercellular exchanges, including growth factors, neuropeptides, neurotransmitters, and cell adhesion molecules, activate intracellular signaling cascades leading to functional and structural changes that promote the formation of functional synapses^1–5^. The synaptic proteins supporting the morphological changes that mediate the formation, restructuring, and elimination of synapses have been extensively studied^1,5^. However, the extracellular factors and the intracellular signal transduction pathways that regulate the expression of the genes encoding proteins required for appropriate synaptic connectivity remain largely unknown.

Over a century ago Santiago Ramón y Cajal proposed that the processes underlying the development of the nervous system and the neural plasticity supporting learning and memory in adulthood share fundamental mechanisms^6^. Indeed, in the last two decades, research examining developmental and learning-related plasticity has uncovered a striking degree of overlap between the molecular mechanisms employed by the brain during these different biological processes^1,4,7–10^. For example, the members of the neurotrophin family of growth factors were initially characterized as critical regulators of neuronal survival, differentiation, and growth^11,12^, but were later shown to contribute to the molecular events underlying activity-dependent synaptic growth and restructuring in the mature nervous system^3,13^. These findings raise important questions about the mechanistic similarities between neural development and adult plasticity, and offer opportunities for integrating findings from both fields of research.

**Figure 1:**
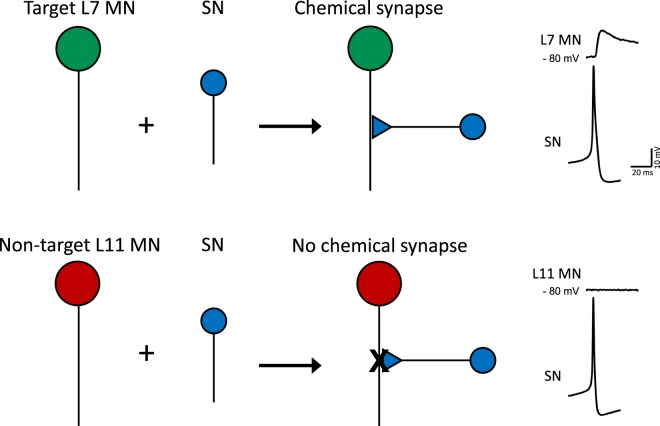
Specificity of synapse formation in *Aplysia* co-cultures: Cultured *Aplysia* SNs selectively form functional chemical synapses with physiological target MNs (L7 MNs), but not with non-target MNs (L11 MNs). The presence of a functional synapse can be tested electrophysiologically by recording evoked postsynaptic potentials: firing of an action potential in the SN elicits an EPSP in the L7 MN, but no response in the L11 MN.

In the present study we tested the general hypothesis that, in the same neural circuit, molecular mechanisms known to regulate learning-related synaptic plasticity also underly the specificity of synapse formation. The marine mollusk *Aplysia californica* is a useful model system for studying the cellular and molecular mechanisms underlying these forms of plasticity^9,14^. In *Aplysia*, facilitation or depression of monosynaptic connections between identified presynaptic sensory neurons (SNs) and postsynaptic motor neurons (MNs) contributes significantly to different forms of learning that can be induced in simple defensive withdrawal reflexes^15,16^. Importantly, this SN-MN microcircuit demonstrates synapse-specificity and can be reconstituted in culture^17^. *In vitro*, as *in vivo, Aplysia* SNs selectively form chemical synapses with their physiological target MNs (L7 MNs), but do not form synapses either with themselves or with non-target MNs (L11 MNs) (Fig. 1)^18,19^. Thus, this *in vitro* system offers single-cell spatial resolution for the examination of the molecular mechanisms underlying the generation of specific neural connections. Previous studies examining the processes underlying the specificity of synapse formation in the *Aplysia* SN-MN co-culture system^19–24^ revealed dynamic interactions between SNs and MNs, suggesting that different cells can “recognize” each other in a selective manner and raising important questions about the nature and mechanisms of the intercellular signaling mediating these effects.

Using neuronal co-cultures, pharmacological manipulations, and single-cell analyses, in the present study we investigated the molecular processes governing the interactions between SNs and target and non-target MNs during the period of synapse formation. We focused our analyses on three evolutionarily-conserved mechanisms that are involved in both developmental and learning-related plasticity: (i) growth factor and neuropeptidergic signaling, (ii) protein kinase activation, and (iii) cyclic-AMP response element binding protein (CREB)-dependent transcription. Our results reveal a surprising bidirectional regulation of molecular signaling between *mismatched* synaptic partners. This regulation is mediated by signaling via both paracrine and autocrine diffusible factors that induce differential effects on transcription and on protein expression/activation in SNs and in target and non-target MNs. Collectively, our data reveal novel molecular mechanisms that could underlie the repression of inappropriate synapse formation.

## Results

### SNs downregulate *C/EBP* expression specifically in non-target MNs

**Figure 2:**
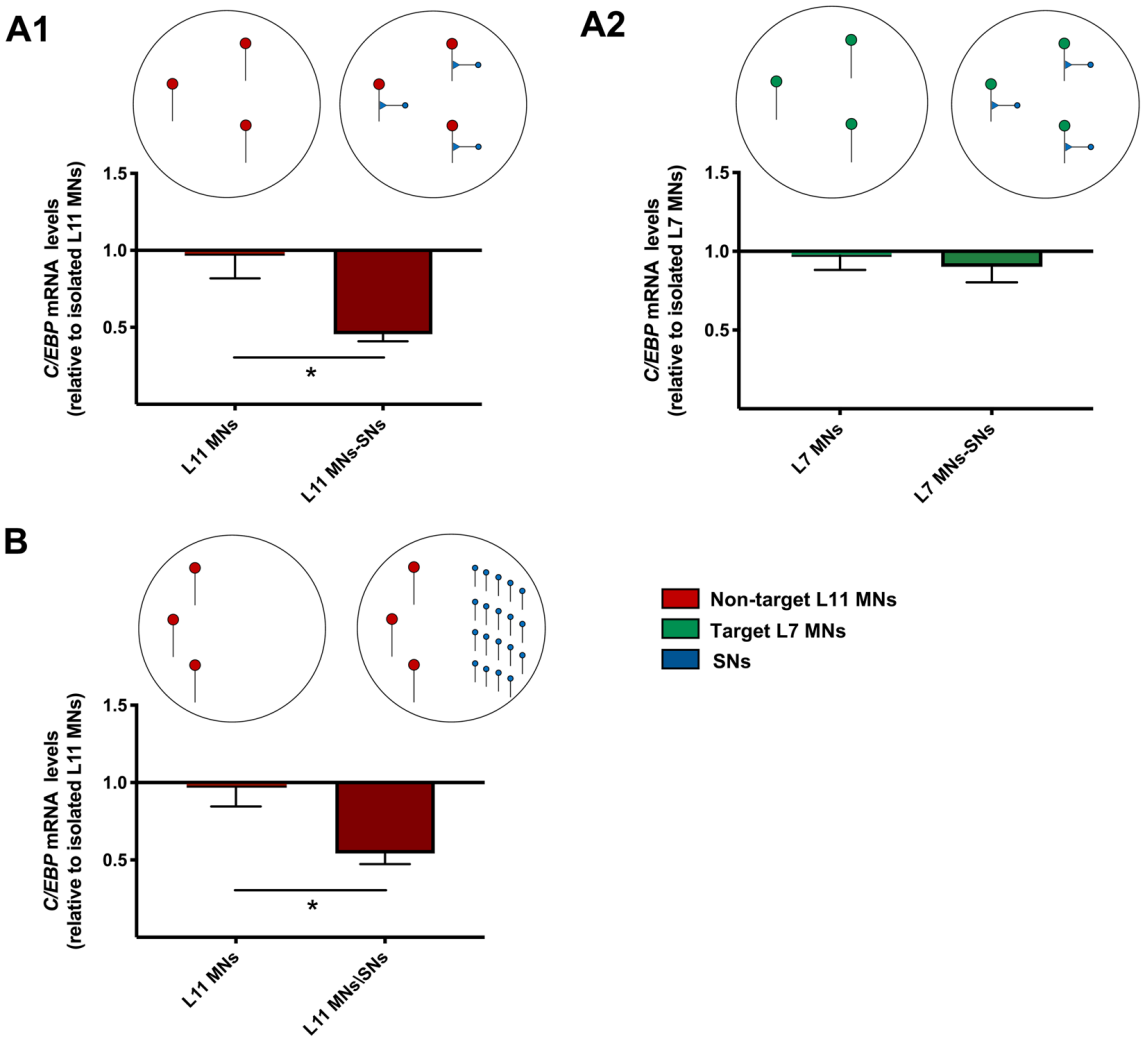
SNs downregulate *C/EBP* expression specifically in non-target MNs through diffusible factor(s): (**A1**) *C/EBP* mRNA levels in non-target L11 MNs paired with SNs were significantly lower than those in isolated L11 MNs. (**A2**) There was no significant difference in *C/EBP* mRNA levels between isolated and paired target L7 MNs. (**B**) *C/EBP* mRNA levels in conditioned non-target L11 MNs were significantly lower than those in isolated L11 MNs. Mean + SEM, *p < 0.05.

We first investigated whether SNs regulate transcriptional events in both non-target and target MNs (Fig. 2). We focused our analysis on gene expression of the transcription factor and immediate-early gene CCAAT-enhancer binding protein (C/EBP). C/EBP is expressed downstream of CREB signaling, a transcriptional pathway that regulates both developmental and learning-related plasticity in *Aplysia* and other systems^25,26^. To explore transcriptional effects of SNs on MNs, we cultured non-target L11 MNs and target L7 MNs in isolation or in contact with SNs (Fig. 2A1,A2). SNs fasciculate with both L11 and L7 MNs, but only form synapses with L7 MNs. After growing for 5 days *in vitro*, we collected the MNs and processed them for single-cell qRT-PCR to monitor mRNA levels. *C/EBP* mRNA levels were normalized to those of the housekeeping gene GAPDH in the same cell, and these normalized values are displayed as fold induction relative to those in isolated MNs. We observed that SNs differentially regulate *C/EBP* mRNA levels in non-target and target MNs. Surprisingly, *C/EBP* mRNA levels in non-target L11 MNs paired with SNs were significantly lower than those in isolated L11 MNs (Fig. 2A1; two-tailed unpaired Student’s *t*-test: Isolated L11 MNs: 1 + 0.18, *n* = 10; Paired L11 MNs: 0.45 + 0.04, *n* = 10; *p* = 0.015, *t*_10_ = 2.901). However, there was no significant difference in *C/EBP* mRNA levels between isolated and paired target L7 MNs (Fig. 2A2; Isolated L7 MNs: 1 + 0.11, *n* = 9; Paired L7 MNs: 0.90 + 0.09, *n* = 8; *p* = 0.539, *t*_15_ = 0.627). These results suggest that SNs downregulate *C/EBP* mRNA levels in non-target MNs, but not in target MNs.

We next explored the molecular mechanisms mediating the observed mRNA regulation between mismatched synaptic partners. We first asked the question: Is physical contact between SNs and non-target MNs necessary for the downregulation of *C/EBP* expression in MNs? To address this question, we cultured non-target L11 MNs either in isolation or in the same culture dish with SNs (conditioned MNs), without physical contact between MNs and SNs (Fig. 2B). We found that *C/EBP* mRNA levels in conditioned non-target L11 MNs were still significantly lower than those in isolated L11 MNs (Fig. 2B; two-tailed unpaired Student’s *t*-test: Isolated L11 MNs: 1 + 0.15, *n* = 7; Conditioned L11 MNs: 0.54 + 0.06, *n* = 6; *p* = 0.026, *t*_11_ = 2.563). These data show that the mRNA regulation exerted by SNs on non-target MNs is not mediated by contact-dependent interaction between neurons, but, rather, is induced through the actions of diffusible factor(s) secreted by SNs.

### A TrkB ligand is required for the SN-induced downregulation of *C/EBP* expression in non-target MNs

**Figure 3:**
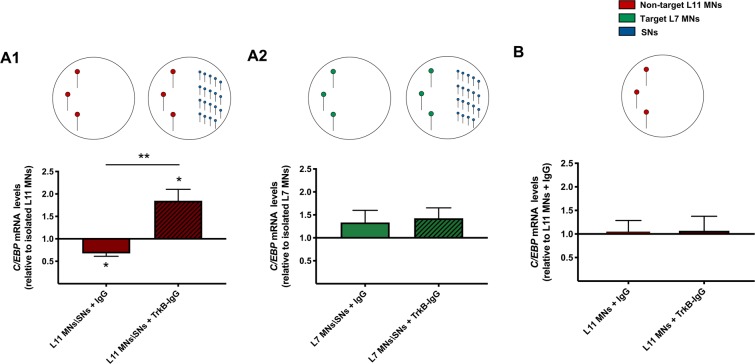
A TrkB ligand is required for the SN-induced downregulation of *C/EBP* expression in non-target MNs: (**A1**) In the presence of IgG, *C/EBP* mRNA levels in conditioned non-target L11 MNs were significantly lower than those in isolated L11 MNs. In the presence of TrkB-IgG, *C/EBP* mRNA levels in conditioned non-target L11 MNs were significantly higher than those in isolated L11 MNs. (**A2**) In target L7 MNs there were no significant differences in *C/EBP* mRNA levels between isolated and conditioned MNs in the presence of IgG or TrkB-IgG. (**B**) TrkB-IgG treatment did not affect *C/EBP* mRNA levels in isolated non-target MNs. Mean + SEM, *p < 0.05, **p < 0.01.

Growth factors are extracellularly released proteins that mediate both developmental and learning-related plasticity^3,4^. Signaling through the tropomyosin receptor kinase B (TrkB), in particular, has been shown to act through the cyclic-AMP pathway to regulate neural development in mammals^27,28^, and long-term synaptic plasticity and memory in both mammals^29,30^ and *Aplysia*^31,32^. The requirement of TrkB signaling for the molecular, cellular, and behavioral expression of long-term memory in *Aplysia* has been shown pharmacologically by blocking endogenous TrkB signaling with soluble TrkB receptor bodies, TrkB-IgG, which sequester secreted BDNF/NT4-like ligands and prevent them from binding to endogenous TrkB-like receptors and inducing intracellular signaling^31,32^. Thus, we used the TrkB-IgG receptor bodies in our experiments to test the hypothesis that a TrkB ligand is mediating the mRNA regulation effects of SNs on non-target MNs. Towards this end we cultured isolated or SN-conditioned non-target and target MNs in the presence of either control- or TrkB-IgG (Fig. 3). We found that *C/EBP* mRNA levels in control IgG-treated conditioned non-target L11 MNs were significantly lower than those in isolated L11 MNs, a replication of our previous results (Fig. 3A1; one-way ANOVA: *F* (2, 15) = 11.25, *p* = 0.001; one-tailed Dunnett’s multiple comparisons test: Isolated L11 MNs: 1 + 0.11, *n* = 11; Conditioned L11 MNs + IgG: 0.67 + 0.06, *n* = 8; *p* = 0.04, *t*_15_ = 2.422). To our surprise, *C/EBP* mRNA levels in TrkB-IgG-treated conditioned non-target L11 MNs were significantly higher than those in isolated L11 MNs (Fig. 3A1; two-tailed Dunnett’s multiple comparisons test: Isolated L11 MNs: 1 + 0.11, *n* = 11; Conditioned L11 MNs + TrkB-IgG: 1.84 + 0.25, *n* = 10; *p* = 0.02, *t*_12_ = 3.025). This pattern of results was specific to non-target L11 MNs, as a one-way ANOVA analysis did not show a significant difference between group means in the target L7 MNs condition (Fig. 3A2, *F* (2, 9) = 2.28, *p* = 0.321; Isolated L7 MNs: 1 + 0.3, *n* = 8; Conditioned L7 MNs + IgG: 1.33 + 0.26, *n* = 8; Conditioned L7 MNs + TrkB-IgG: 1.42 + 0.22, *n* = 7). Importantly, the effects of TrkB-IgG treatment were specific to conditioned non-target L11 MNs, and were not observed in isolated non-target L11 MNs (Fig. 3B; two-tailed unpaired Student’s *t*-test: Isolated L11 MNs + IgG: 1 + 0.28, *n* = 6; Isolated L11 MNs + TrkB-IgG: 1.06 + 0.3, *n* = 6; *p* = 0.874, *t*_10_ = 0.162). Collectively, these results reveal three features of the engagement of TrkB signaling in the SNs-induced regulation of *C/EBP* expression in MNs: (i) TrkB signaling is required for SNs-induced *C/EBP* downregulation in non-target MNs; (ii) in the absence of TrkB signaling, SNs upregulate *C/EBP* expression in non-target MNs, but not in target MNs; and (iii) TrkB signaling-dependent regulation of *C/EBP* mRNA levels in non-target MNs is contingent on signaling from SNs.

### Sensorin upregulates *C/EBP* expression specifically in non-target MNs, but only in the absence of autocrine TrkB signaling

**Figure 4:**
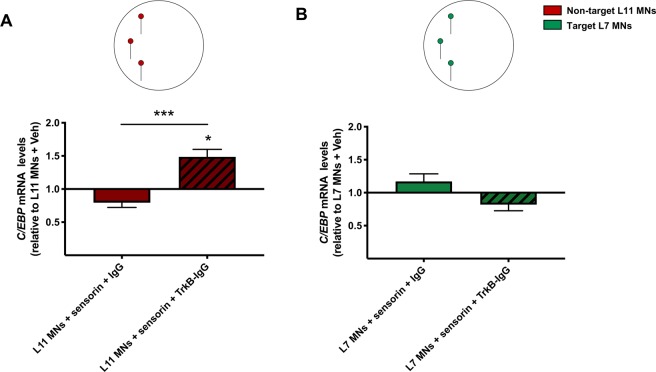
Sensorin upregulates *C/EBP* expression specifically in non-target MNs, but only in the absence of autocrine TrkB signaling: (**A**) Treatment with sensorin peptide and control IgG did not significantly regulate *C/EBP* expression in isolated non-target L11 MNs. In the presence of TrkB-IgG, sensorin significantly upregulated *C/EBP* expression in isolated non-target L11 MNs. (**B**) In target L7 MNs, sensorin did not significantly regulate *C/EBP* expression in the absence or presence of TrkB-IgG. Mean + SEM, *p < 0.05, ***p < 0.001.

Blocking TrkB signaling not only blocked the SNs-induced *C/EBP* downregulation in non-target MNs, but, to our surprise, also revealed an independent diffusible signaling pathway from SNs that facilitates *C/EBP* expression in the absence of TrkB signaling. Sensorin is a SN-specific secreted neuropeptide in *Aplysia* that has been shown to play critical roles in both synapse formation between SNs and target L7 MNs^21,22,24^, and in memory-related synaptic plasticity^24,33–35^. Thus, we tested the hypothesis that sensorin signaling mediates the SN-induced *C/EBP* regulation in non-target MNs. In these experiments, isolated non-target L11 MNs or target L7 MNs were cultured in the presence of (i) vehicle, (ii) sensorin peptide, and (iii) control- or TrkB-IgG. Treatment with sensorin peptide did not have a significant effect on *C/EBP* expression in isolated non-target L11 MNs (Fig. 4A; one-way ANOVA: *F* (2, 28) = 12.9, *p* = 0.001; two-tailed Dunnet’s multiple comparisons test: L11 MNs + vehicle: 1 + 0.13, *n* = 20; L11 MNs + sensorin + IgG: 0.79 + 0.07, *n* = 18; *p* = 0.456, *t*_28_ = 1.349). However, in the presence of TrkB-IgG, treatment with sensorin peptide significantly upregulated *C/EBP* expression in isolated non-target L11 MNs (Fig. 4A; L11 MNs + vehicle: 1 + 0.13, *n* = 20; L11 MNs + sensorin + TrkB-IgG: 1.48 + 0.11, *n* = 13; *p* = 0.028, *t*_30_ = 2.761). Moreover, there was a significant difference in the effects of sensorin on *C/EBP* expression between control IgG- and TrkB-IgG-treated L11 MNs (*p* = 0.0001, *t*_21_ = 5.139). As predicted from the lack of SN regulation of *C/EBP* expression in target L7 MNs, one-way ANOVA analysis did not show significant differences between the vehicle, sensorin, and control or TrkB-IgG-treated groups in the target L7 MNs condition (Fig. 4B, *F* (2, 29) = 2.872, *p* = 0.07; L7 MNs + vehicle: 1 + 0.1, *n* = 17; L7 MNs + sensorin + IgG: 1.17 + 0.11, *n* = 16; L7 MNs + sensorin + TrkB-IgG: 0.81 + 0.09, *n* = 14). These findings indicate that sensorin signaling alone is not sufficient to significantly downregulate *C/EBP* mRNA levels in the presence of TrkB signaling in non-target MNs (although it induces a trend towards downregulation similar to that observed with signaling from SNs). Interestingly, sensorin signaling is sufficient to induce *C/EBP* upregulation in the absence of TrkB signaling in non-target MNs, an effect comparable to that observed in non-target MNs cultured in the presence of SNs (Fig. 3A1). Furthermore, the experiments on isolated non-target MNs show that the TrkB ligand is released from the non-target MNs themselves and acts in an autocrine manner to reverse the facilitatory mRNA regulation effects of sensorin signaling.

### Sensorin upregulates MAPK activation specifically in non-target MNs

**Figure 5:**
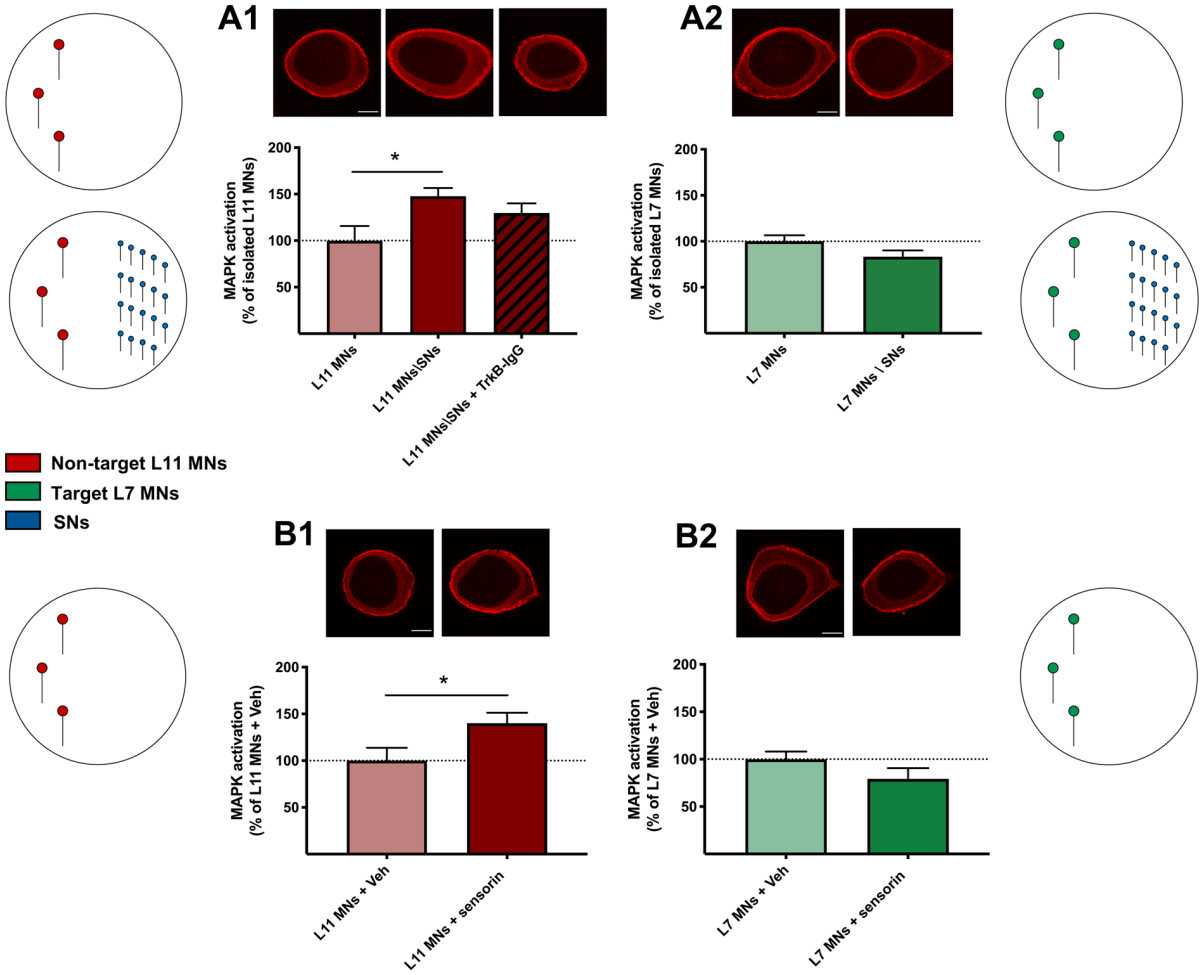
Sensorin upregulates MAPK activation specifically in non-target MNs: (**A1**) MAPK activation was significantly increased in conditioned over isolated non-target L11 MNs, and treatment with TrkB-IgG did not significantly disrupt this activation. (**A2**) There was no significant difference in MAPK activation between isolated and conditioned target L7 MNs. (**B1**) Treatment with sensorin peptide induced significant MAPK activation in isolated non-target L11 MNs, (**B2**) but not in isolated target L7 MNs. Mean + SEM, *p < 0.05. Scale bar 25 μm.

We next investigated a signal transduction mechanism that could be upstream of the mRNA regulation events we observed thus far. The activation of mitogen-activated protein kinase (MAPK), a well-characterized second messenger cascade that links extracellular signaling to transcriptional events in the nucleus^36^, is required for both developmental and memory-related plasticity^37,38^. In *Aplysia*, long-term facilitation of SN-MN synapses induces MAPK activation and nuclear translocation^39^, and sensorin signaling has been shown to act upstream of these events^34^. Hence, we focused our next analysis on MAPK activation using immunofluorescence and phospho-specific MAPK antibodies in cultured MNs. We measured the cytoplasmic mean fluorescence intensity of dually-phosphorylated MAPK in the cell body and represented the experimental groups as a percentage of controls. We first tested: (i) whether SNs affected MAPK activation in non-target and target MNs and (ii) if so, whether TrkB signaling was engaged. Our results showed a significant increase in MAPK activation in conditioned non-target L11 MNs when compared with isolated L11 MNs (Fig. 5A1; one-way ANOVA: *F* (2, 26) = 4.037, *p* = 0.029; two-tailed Tukey’s multiple comparisons test: Isolated L11 MNs: 100 + 15.7%, *n* = 10; Conditioned L11 MNs: 147 + 8.5%, *n* = 10; *p* = 0.024, *t*_26_ = 3.981). While modestly reduced, this effect was not significantly disrupted by treatment with TrkB-IgG, suggesting that TrkB signaling does not play a major role in the SN-induced MAPK activation in non-target MNs (Conditioned L11 MNs: 147 + 8.5%, *n* = 10; Conditioned L11 MNs + TrkB-IgG: 129 + 10.2%, *n* = 9; *p* = 0.563, *t*_26_ = 1.460). Furthermore, there was no significant difference in MAPK activation between isolated and conditioned target L7 MNs (Fig. 5A2; two-tailed unpaired Student’s *t*-test: Isolated L7 MNs: 100 + 6.6%, *n* = 10; Conditioned L7 MNs: 83 + 6.9%, *n* = 15; *p* = 0.221, *t*_23_ = 1.667). Collectively, these results show that SNs induce an increase in MAPK activation in non-target MNs, but not in target MNs.

We next examined whether sensorin signaling mediates the SN-induced MAPK activation in non-target MNs. To address this question, we cultured isolated non-target and target MNs in the presence of vehicle or sensorin peptide. Treatment with sensorin peptide led to a significant increase in MAPK activation in non-target L11 MNs (Fig. 5B1; two-tailed unpaired Student’s *t*-test: L11 MNs + vehicle: 100 + 13.8%, *n* = 15; L11 MNs + sensorin: 140 + 11.1%, *n* = 12; *p* = 0.038, *t*_25_ = 2.184), but not in target L7 MNs (Fig. 5B2; L7 MNs + vehicle: 100 + 8.1%, *n* = 14; L7 MNs + sensorin: 79 + 11.2%, *n* = 13; *p* = 0.144, *t*_25_ = 1.508). Taken together, these results indicate that sensorin secreted by SNs induces an increase in MAPK activation specifically in non-target MNs.

### Non-target MNs upregulate sensorin protein levels in SNs

**Figure 6:**
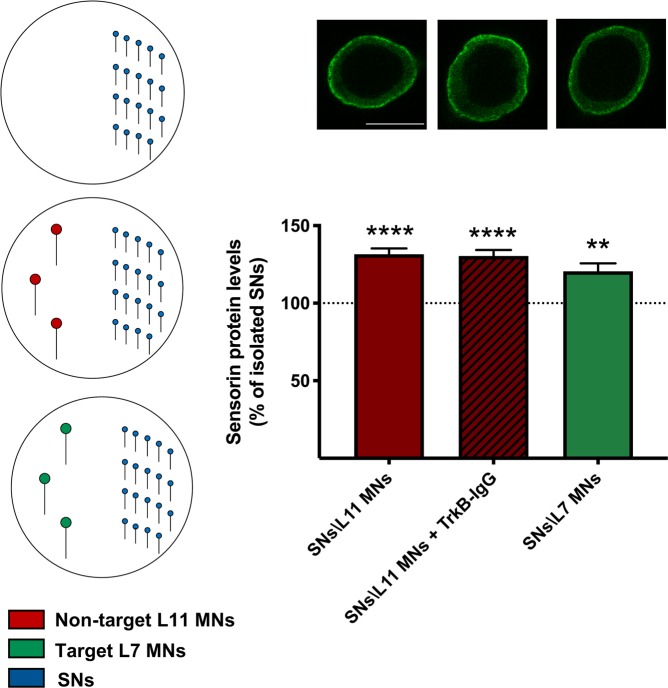
Non-target MNs upregulate sensorin protein levels in SNs: sensorin protein levels were significantly higher in SNs cultured in the presence of non-target L11 MNs, and this increase was not affected by treatment with TrkB-IgG. Sensorin protein levels were significantly higher in SNs cultured in the presence of target L7 MNs. Mean + SEM, **p < 0.01, ****p < 0.0001. Scale bar 25 μm.

Our data thus far show that SNs induce both mRNA downregulation and protein kinase activation specifically in non-target MNs. In a final set of experiments, we investigated the converse, examining whether non-target and target MNs induce molecular changes in SNs. Given our previous findings, we examined changes in sensorin protein levels using immunofluorescence in SNs cultured in isolation, or in the presence of non-target or target MNs. We found that cytoplasmic sensorin protein levels were significantly increased in SNs cultured in the presence of non-target L11 MNs when compared with isolated SNs (Fig. 6; one-way ANOVA: *F* (3, 75) = 12.34, *p* < 0.0001; two-tailed Tukey’s multiple comparisons test: Isolated SNs: 100 + 3.5%, *n* = 22; SNs + L11 MNs: 131 + 3.7%, *n* = 20; *p* < 0.0001, *t*_75_ = 7.68), and this increase was not blocked by treatment with TrkB-IgG (Isolated SNs: 100 + 3.5%, *n* = 22; SNs + L11 MNs + TrkB-IgG: 130 + 3.9%, *n* = 15; *p* < 0.0001, *t*_75_ = 6.835). Interestingly, sensorin protein levels were also significantly higher in SNs cultured in the presence of target L7 MNs when compared with isolated SNs (Isolated SNs: 100 + 3.5%, *n* = 22; SNs + L7 MNs: 120 + 5.1%, *n* = 22; *p* = 0.003, *t*_75_ = 5.115). These data suggest that both non-target and target MNs upregulate sensorin protein levels in SNs.

## Discussion

**Figure 7:**
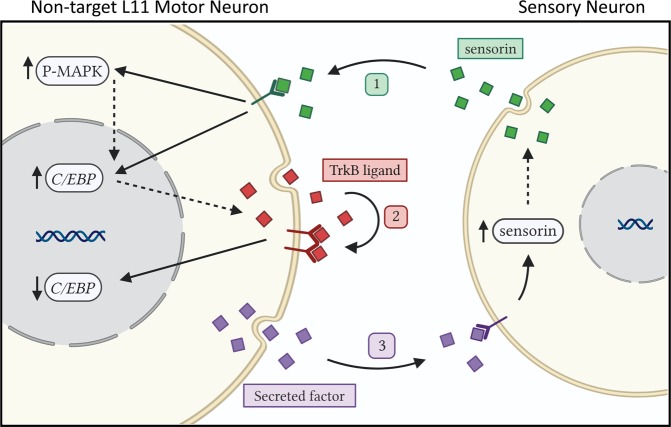
Proposed model of paracrine and autocrine signaling between *mismatched* SNs and MNs.

In the present study we investigated the molecular interactions between SNs and their appropriate and inappropriate target MNs. Our results reveal an unexpected and complex bidirectional regulation of intracellular signaling between *mismatched* synaptic partners. The regulation induced by presynaptic SNs on non-target MNs is governed by (i) paracrine neuropeptidergic signaling from SNs (Figs. 4,5) and (ii) by autocrine growth factor signaling from MNs (Figs. 3, 4). These forms of signaling have facilitatory effects on MAPK activation (Fig. 5), and both facilitatory and inhibitory effects on CREB-dependent transcription in non-target MNs (Figs. 3, 4). In addition, we found evidence for molecular regulation induced by both target and non-target MNs on SNs, which is governed by distinct paracrine signaling pathway(s) from MNs and has facilitatory effects on neuropeptide expression in SNs (Fig. 6). Based on these results, we propose a working model shown in Fig. 7. The model posits at least three pathways of paracrine and autocrine signaling between *mismatched* SNs and MNs. We hypothesize that these processes could collectively help prevent the formation of inappropriate synaptic connections. The model has three basic steps. (1) SNs secrete the neuropeptide sensorin, which binds to receptors on non-target MNs and induces an increase in both MAPK activation and *C/EBP* expression (these two effects could be related, since increased *C/EBP* expression could be downstream of MAPK activation). (2) In response to sensorin signaling (and possibly other signaling molecules from SNs), non-target MNs secrete a TrkB ligand which acts in an autocrine manner to downregulate *C/EBP* expression (increased transcription and/or secretion of the TrkB ligand could be downstream of sensorin-induced *C/EBP* upregulation, creating a negative feedback loop for *C/EBP* expression). (3) An unidentified secreted factor from non-target MNs binds to receptors on SNs and induces an increase in sensorin protein levels; this MN-induced increase in sensorin levels can, in turn, initiate and/or prolong the sensorin secretion in Step (1), creating a positive feedback loop for sensorin signaling. This model of intercellular and intracellular molecular regulation between *mismatched* synaptic partners reveals novel and evolutionarily-conserved molecular mechanisms that could underlie the specificity of synapse formation in this *Aplysia* neural circuit, as well as in other neural systems.

Previous studies in *Aplysia* have shown that SNs and target L7 MNs form functional chemical synapses *in vitro* within a few hours from culturing^17^. The formation of these synapses requires new translation of sensorin mRNA^22,24^ and L7 MN-dependent release of sensorin peptide from SNs^21^, and is accompanied by morphological changes in presynaptic SNs, such as neurite outgrowth and the development of active zones^19^. Interestingly, non-target L11 MNs can also induce some of these changes in SNs, although to an intermediate level between that observed in isolated SNs and in SNs paired with target L7 MNs^19,21,22,24^, indicating that retrograde signaling from both target and non-target MNs can affect SNs. In the present study, we found evidence for both target and non-target MN-induced increases in sensorin protein levels, suggesting that these two types of MNs can have similar molecular effects on SNs. Although our experiments do not reveal the specific identity of the ligand(s) and receptors mediating these effects, they do show that they are independent of TrkB signaling, at least for the non-target MN effects on SNs. Potential candidates for the observed retrograde signal(s) that increase(s) sensorin protein levels in SNs are the netrin family of chemotropic factors. Interestingly, netrin-1 was shown to promote translation of *sensorin* mRNA in the neurites of SNs contacting both target and non-target MNs in *Aplysia*^24^, and to induce synaptogenesis in *C. elegans*^40^. Taken together with previous studies in *Aplysia*^19,21,22,24^, our results strengthen the existing evidence for retrograde diffusible signaling from both target and non-target MNs, and describe similar molecular changes induced by the two types of MNs in SNs. It would be interesting for future studies to investigate whether the diffusible signaling molecules from L11 and L7 MNs are the same and whether their actions depend on signaling from SNs.

Previous research examining the processes underlying the specificity of synaptogenesis in the *Aplysia* co-culture system has reported changes in the cellular distribution of sensorin mRNA in SNs paired with MNs^20–22,24^. For example, an important series of studies showed that both target L7 and non-target L11 MNs induced increases in the expression of sensorin mRNA in the distal neurites of SNs above levels in isolated SNs, although the L7 MN effect was significantly greater than that of the L11 MN^20–22^. Furthermore, downregulation of sensorin mRNA in SNs has been shown to strongly inhibit synapse formation between SNs and target MNs^22^, which requires local translation of sensorin mRNAs^22,24^. In addition, L7 MN-dependent release of sensorin from SNs was found to be required for the regulation of sensorin mRNA distribution as well as for synapse formation^21^. Interestingly, the differences in sensorin mRNA localization between target and non-target MNs were observed after 4 days in culture, but were not detected at earlier time points^20,22^. Moreover, sensorin secretion was reported to decline from day 1 to day 4 in culture^21^. These reports suggest that, although sensorin is secreted from SNs during the early stages of synapse development *in vitro*, the differences in intracellular events between L7 and L11 MNs, such as *sensorin* mRNA distribution, are not present until the later stages of synapse maintenance, highlighting changes in the temporal dynamics of sensorin signaling. While synapse formation between *matched* SNs and MNs occurs during the first hours in culture^17,18,22^, the prevention of synapse formation might engage a prolonged, active process of molecular inhibition between *mismatched* synaptic partners. In the present study we found that, after 5 days in culture, both target and non-target MNs induce significant increases in somatic sensorin protein levels in co-cultured SNs. Thus, our current findings complement and extend previous studies by showing that diffusible factor(s) secreted from MNs can act over long spatial and temporal ranges to regulate sensorin expression in SNs, even in the absence of physical contact between neurons. It would be interesting for future studies to investigate whether the increase in somatic sensorin protein expression we observed is due to *de novo* transcription and/or translation, or alternatively, is due to decreased secretion or transport to the neurites.

Whether SNs have any effects on MNs, on the other hand, is a less studied question. A study in *Aplysia*^23^ investigated the differences in synaptic protein expression between L7 and L11 MNs and found that both types of MNs expressed comparable basal levels of AMPA- and NMDA-like receptors and of the Dscam protocadherins family of cell adhesion molecules (CAMs). Moreover, culturing SNs with target L7 MNs induced Dscam-dependent clustering of both AMPA and NMDA receptors on the cell surface of L7 MNs. Interestingly, only clustering of NMDA, but not of AMPA receptors, was observed in L11 MNs that were co-cultured with SNs^23^. CAMs are critical for the trans-synaptic signaling underlying synaptogenesis, and their transcription is tightly regulated during this process^1,5^. In addition, postsynaptic CAMs, such as latrophilins and cadherins, have been reported to mediate synapse specificity^41–43^. In *Aplysia*, the postsynaptic protocadherins Dscam were found to be required for *de novo* synaptogenesis between SNs and L7 MNs and for AMPAR clustering in L7 MNs^23^. Collectively, these findings suggest the intriguing possibility that L11 MNs express the receptors necessary for glutamatergic synapse formation with SNs, and that an active downregulation process acts to prevent AMPAR clustering in these inappropriate MNs. Differential transcription regulation of CAMs in target and non-target MNs could lead to differences in AMPAR clustering and thus could underlie synapse specificity. Our current findings that signaling from SNs downregulates CREB-dependent transcription in non-target MNs could provide a possible transcriptional mechanism through which CAM-dependent AMPAR clustering is inhibited in non-target MNs.

The transcription and expression of synaptic proteins such as CAMs and neurotransmitter receptors can affect the structural and functional architecture of neural connections^1^. In the present study we investigated the functional significance of reversing the SN-induced *C/EBP* downregulation in non-target MNs. We tested electrophysiologically the presence of functional chemical synapses between SNs and target and non-target MNs treated with control- or TrkB-IgG (Fig. S1). 100% of tested SNs - L7 MNs synapses exhibited excitatory postsynaptic potentials (EPSPs), while none of the recordings in control IgG- or TrkB-IgG-treated SNs - L11 MNs pairs revealed postsynaptic responses. These results indicate that blocking the *C/EBP* downregulation effects of TrkB signaling is not sufficient to promote functional synapse formation between SNs and non-target MNs. Moreover, these experiments show that the 5-day treatment with TrkB-IgG does not affect the electrophysiological properties of L11 MNs or of SNs (Fig. S1). It would be important for future research to investigate whether a reversal of *C/EBP* downregulation through TrkB signaling blockade has an effect on the expression of synaptic proteins in non-target L11 MNs and whether additional steps such as neural activity are required to promote synapse formation between SNs and non-target MNs.

Our results show that, in the absence of TrkB signaling, exogenous application of sensorin peptide to isolated non-target MNs, recapitulates the facilitatory MAPK activation and *C/EBP* mRNA expression effects induced by SNs in non-target MNs. Interestingly, our data suggest that TrkB signaling acts in opposition to sensorin signaling, and most likely downstream of it, to downregulate *C/EBP* expression. Taken together with previous studies, our findings support a model whereby sensorin signaling acts during the early stages of synapse development in *Aplysia* co-cultures to facilitate synaptogenesis, while TrkB signaling acts during the later stages of this process to repress inappropriate synapse formation. Thus, sensorin could be secreted from SNs in a general manner, and induce differential effects in target and non-target MNs. In response to sensorin and possibly other signaling from SNs, autocrine TrkB signaling in non-target MNs could induce a long-lasting transcriptional repression in non-target MNs, thus providing an inhibitory constraint on inappropriate synapse formation. BDNF, one of the mammalian TrkB ligand orthologs, has been shown to act in an autocrine manner to stabilize cyclic AMP-dependent axon initiation and growth^28^. Interestingly, BDNF can act through TrkB receptors to promote neural development and synaptic strengthening, and through the p75NTR receptor to promote neuronal death and synaptic depression^10^. Moreover, reports from the mammalian and invertebrate literature have shown that the same growth factor can both promote and inhibit synaptogenesis by signaling through distinct receptors and inducing different downstream signaling^44–47^. The soluble receptor bodies we used in our experiments to sequester endogenously released TrkB ligands do not reveal the identity of the endogenous receptor whose signaling they disrupt. Hence, the TrkB ligand sequestered by TrkB-IgG could, in principle, act through an endogenous p75NTR receptor^48^ to induce downregulation of CREB-dependent transcription in L11 MNs. Taken together, our findings suggest that (i) long-range diffusible signaling through the paracrine actions of sensorin neuropeptide and of unidentified factor(s) secreted by MNs, and (ii) local signaling through the autocrine actions of a TrkB ligand in non-target MNs, could induce permissive and non-permissive states of transcription in target and non-target MNs, respectively, thereby providing target recognition signals that could underlie the specificity of synapse formation.

Importantly, our data describing the regulation of *C/EBP* mRNA levels do not address whether the observed regulation represents *de novo* gene expression or posttranscriptional regulation of mRNA stability^49^, both of which have been shown to be downstream of MAPK activation^38,50^. However, since C/EBP is a transcription factor, the regulation of its mRNA will most likely result in altered transcription of downstream late-response genes that, in turn, could alter the synaptic architecture of target and non-target MNs.

In conclusion, the results in the present study reveal novel intercellular and intracellular molecular mechanisms underlying the interactions between cultured *Aplysia mismatched* synaptic partners. Our findings provide evidence highlighting the differences and similarities between the processes mediating the promotion of appropriate neural connections and the prevention of inappropriate ones. Future work investigating earlier time points in this culture system are likely to reveal facilitatory effects of SNs on target MNs that might be mediated by similar signaling mechanisms with the ones we describe here. While little is known about the processes involved in the initial formation of synapses between SNs and MNs during development in this system, our study revealing mechanistic aspects of specificity of synapse formation between cultured adult neurons can inform future developmental research. Furthermore, the evolutionarily-conserved molecular mechanisms we investigated in the context of synaptogenesis, such as TrkB and sensorin signaling, MAPK activation, and CREB-dependent transcription, are known to be engaged during learning-related synaptic plasticity in the same and other systems^3,4,25,26,32,34,35,39^. While clear similarities between the two programs of plasticity have been previously described^7–10^ and were suggested by our present findings (e.g., activation of MAPK and of TrkB and sensorin signaling), our results also revealed differences in the valence of transcriptional effects (e.g., *C/EBP* downregulation vs. upregulation in learning). Taken together with previous research in *Aplysia*, the present study reveals novel molecular mechanisms of plasticity that could represent points of contact between developmental synapse formation and experience-dependent synaptic restructuring.

## Methods

### Animals and cell culture

Adult (70–90 g) and juvenile (1–4 g) *Aplysia californica* from the *National Resource for Aplysia* Facility at University of Miami were housed in an aquarium for at least 3 days before experimentation. Pleural SNs and abdominal L11 and L7 MNs were identified based on morphological characteristics and location. SNs and MNs were cultured according to a published protocol^17^ and kept in culture for 5 days. SN-MN pairs consisted of a single MN paired with a single SN (3–4 pairs per plate). Plates with isolated or conditioned neurons consisted of 3–5 MNs cultured in isolation or in the presence of ~30 SNs (conditioned neurons were cultured with ~3 mm distance between SNs and MNs).

### Pharmacological treatments

TrkB-IgG or control IgG (2 μg/ml; R&D Systems) was added to cultured MNs 20 min before the addition of SNs or sensorin to the culture plates, and was left in the culture medium until RNA extraction on day 5. Sensorin peptide (500 ng/ml; CTRSKNNVPRRFPRARYRVGYMF; Genemed Synthesis) or Vehicle (0.1% BSA in PBS) was added to isolated MNs on the day of culture and was left in the culture medium until RNA extraction on day 5.

### RNA isolation, cDNA synthesis, and qRT-PCR

Total RNA from single MNs was isolated and purified using the RNAqueous-Micro Total RNA Isolation Kit (Invitrogen). cDNA was synthesized using the SuperScript IV Reverse Transcriptase Kit (Invitrogen). Quantitative PCR was performed using a Roche LightCycler 480 and SybrGreen I Master (Roche; 5 min at 95 °C, 40 cycles of: 10 sec at 95 °C, 15 sec at 56 °C, and 15 sec at 72 °C). The following primers were used: ApGAPDH (F-5′-CTCTGAGGGTGCTTTGAAGG-3′; R-5′-GTTGTCGTTGAGGGCAATTC-3′), ApC/EBP (F-5′-TACGTGGATAAGAGGGCCAGA-3′; R-5′-GACTTCACACGACCCTCTGTT-3′). The amount of each gene was normalized to that of ApGAPDH within the same sample using the ΔΔCt method.

### Immunofluorescence

Cultured neurons were fixed for 30 min in a solution of 4% formaldehyde in TBS containing 30% sucrose. After 5 washes with TBS, fixed cells were blocked for 30 min at room temperature in a TBS blocking solution containing 10% normal goat serum and 0.2% Triton X-100. Cells were then incubated for 3 nights at 4 °C with anti-sensorin (1 μg/ml; custom-made) or anti-phosphorylated MAPK antibody (1:200; Cell Signaling Technology). After 3 washes with TBS, a secondary antibody conjugated to Cy-5 (1:500; Abcam) was applied for 2 hrs at room temperature. Cells were then washed 4 times in TBS and mounted using ProLong Gold Antifade Mountant (Invitrogen). Images were obtained with a Leica SP8 confocal microscope using a 63 X oil-immersion lens. Images taken through the middle of the nucleus were used for ImageJ analysis of mean fluorescence intensity in the cell body.

### Electrophysiology

Electrophysiological recordings were performed in current-clamp mode on SN-MN co-cultures. Extracellular stimulation of SNs was performed using blunt glass microelectrodes filled with ASW. Intracellular recordings from MNs were performed using 5–12 MΩ glass microelectrodes filled with 3 M KCl. MNs were held at approximately −80 mV by passing constant hyperpolarizing current. SNs were stimulated to elicit a single action potential, and excitatory postsynaptic potentials (EPSPs) were recorded in the follower MNs.

### Statistical analyses

All analyses were conducted using GraphPad Prism. All data are expressed as mean + SEM. Parametric statistics were used throughout due to a normal distribution of the data. For two-group comparisons unpaired Student *t*-tests were used. For comparisons of three or more groups, one-way ANOVA followed by planned multiple comparisons tests were used. qPCR data was analyzed using Dunnett’s multiple comparisons tests due to unequal variance between groups. Immunofluorescence data was analyzed using Tukey’s multiple comparisons test due to equal variance between groups. All *p* values are reported for two-tailed analysis, unless otherwise specified. Statistical significance is defined as *p < 0.05, **p < 0.01, ***p < 0.001, ****p < 0.0001.

### Ethical approval and informed consent

The experiments in this study do not involve human subjects or samples from humans, other vertebrates or higher invertebrates.

## Supplementary information

Supplementary Figure S1

## Data Availability

Materials, data, and associated protocols generated during the current study are available from the corresponding author on reasonable request.
