# Supplementary material for: Specificity of synapse formation in Aplysia: paracrine and autocrine signaling regulates bidirectional molecular interactions between sensory and non-target motor neurons
Source: Sci Rep. 2020 Mar 23;10:5222. doi: 10.1038/s41598-020-62099-4 (PMC7089980; doi:10.1038/s41598-020-62099-4)
Supplement: Supplementary file 1 — Supplementary Figure S1 [file 41598_2020_62099_MOESM1_ESM.pdf]

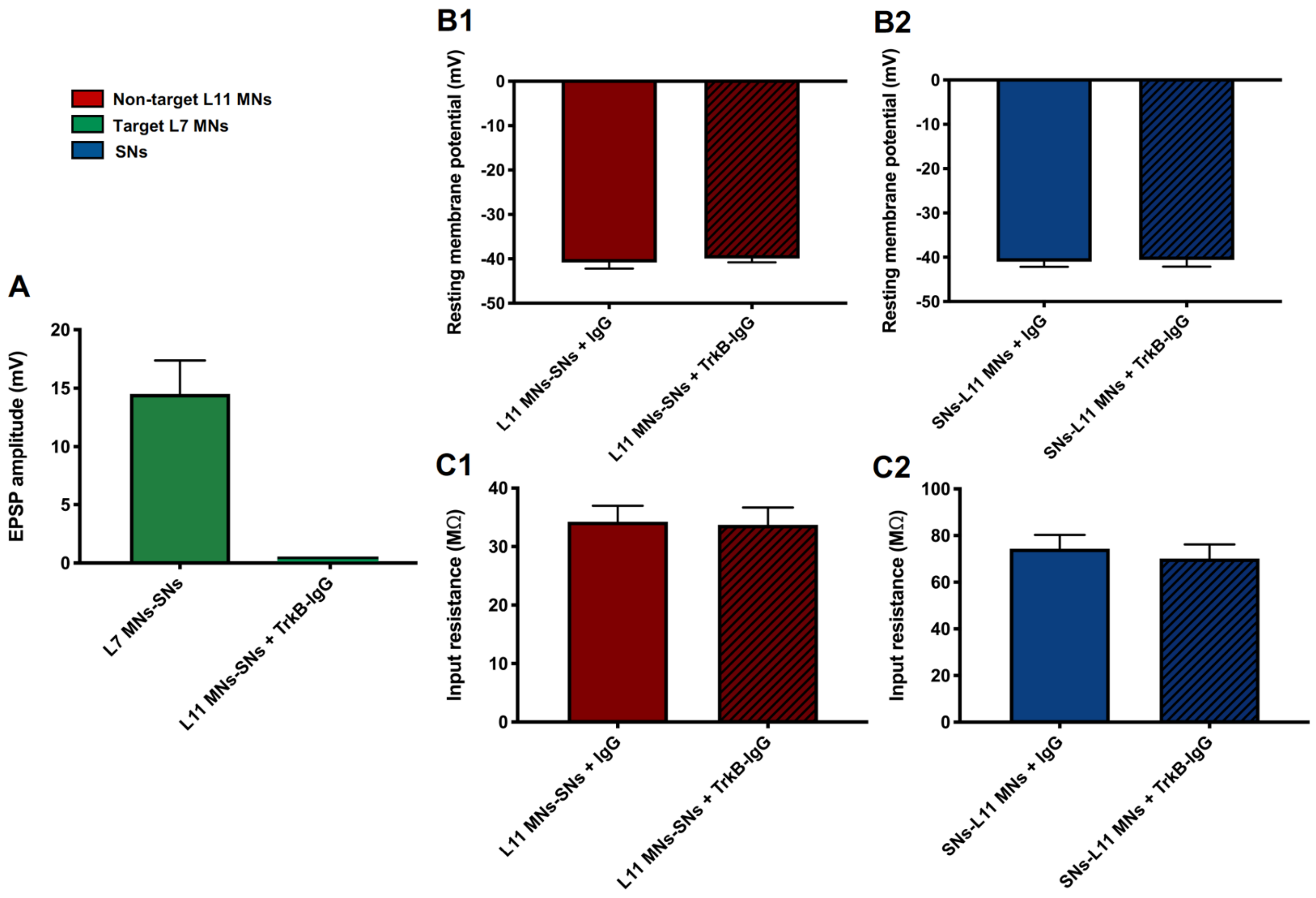

Supplementary figure 1. Blocking TrkB signaling does not induce formation of functional synapses between SNs and non-target MNs. (A) 100% of paired L7 MNs exhibited EPSP (14.5 + 2.8 mV, *n* = 8), but none of the TrkB-IgG-treated paired L11 MNs did (0 mV, *n* = 7). **(B1)** TrkB-IgG did not affect the resting membrane potential of L11 MNs (IgG: -40.8 + 1.3 mV, *n* = 8; TrkB-IgG: -39.9 + 0.8, *n* = 7; *p* = 0.594) or of **(B2)** SNs (IgG: -41 + 1.1 mV, *n* = 8; TrkB-IgG: -40.6 + 1.4, *n* = 7, *p* = 0.844). **(C1)** TrkB-IgG did not affect the input resistance of L11 MNs (IgG: 34.2 + 2.7 MΩ, *n* = 8; TrkB-IgG: 33.7 + 2.9 MΩ, *n* = 7, *p* = 0.896) or of **(C2)** SNs (IgG: 74.3 + 5.8 MΩ, *n* = 8; TrkB-IgG: 70.1 + 6.1 MΩ, *n* = 7, *p* = 0.624). Mean + SEM, two-tailed unpaired Student's *t*-tests.
